# Half a century of evolutionary games: a synthesis of theory, application and future directions

**DOI:** 10.1098/rstb.2021.0492

**Published:** 2023-05-08

**Authors:** Xiang-Yi Li Richter, Jussi Lehtonen

**Affiliations:** ^1^ Institute of Biology, University of Neuchâtel, Rue Emile-Argand 11, 2000 Neuchâtel, Switzerland; ^2^ Department of Biological and Environmental Science, University of Jyväskylä, 40014 Jyväskylä, Finland

Evolutionary game theory, with its explicit incorporation of frequency-dependent selection into evolutionary thinking, was one of the great leaps forward since Darwin's insight [1]. Adaptation via natural selection is intuitive and straightforward to envision in static, abiotic environments: whales and fishes look as if they were designed to function in water; birds and bats seem to be designed for airborne life. Evolutionary game theory, on the other hand, provides a powerful toolkit and conceptual framework for modelling adaptation for biotic interactions: in many cases, the adaptive environment consists of the evolving population itself, which is far from static and brings with it a different way of modelling evolution and its outcomes.

Game theory can therefore be used to solve problems where the pay-off for adopting a given strategy depends on the strategies adopted by others, and there is no obvious ‘best thing’ for an individual to do. This underlying idea applies whether the focus is on economic behaviour in humans, or on evolution in populations of organisms that do not necessarily have the capacity for rational thinking. Although the formal origins of game theory lie in economic behaviour, in hindsight, it does not seem surprising that the leap to evolution would be made: in his classic textbook [2, p. vii] Maynard Smith wrote: ‘Paradoxically, it has turned out that game theory is more readily applied to biology than to the field of economic behaviour for which it was originally designed’, the main reason being that no assumption of a ‘rational actor’ is needed. In hindsight, then, the core of evolutionary game theory has an air of being ‘obvious’, similar to Darwin's discoveries. Furthermore, we typically do not need to know the details of genetics to work with evolutionary game theory, just as Darwin did not know of genetics in his time. Indeed, it has been claimed that Darwin would have loved evolutionary game theory, had he been aware of it [3].

Published 50 years ago, ‘The logic of animal conflict’ by John Maynard Smith & George Price [4] is the paper that is typically considered the ‘birth’ of evolutionary game theory (though there were strong precedents, especially in the field of sex ratio theory [5]). Leimar & McNamara [6] and Grodwohl & Parker [7] provide a comprehensive discussion of the historic value of Maynard Smith & Price [4]. The article of Maynard Smith and Price was largely motivated by the prevalence of ‘limited war’ conflicts in nature. Why do fights in nature not more commonly escalate far enough to result in serious injury or death? The computer simulations in the original work of Maynard Smith & Price [4] were later summarized and simplified into a simple toy model, called the Hawk-Dove game (see [6]). This game describes the interactions of two strategies in a population of players. The pay-off matrix is as follows:


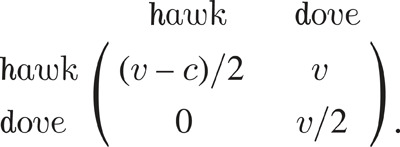


In the pay-off matrix, *v* stands for the value of the resource and *c* stands for the cost of an escalated fight. It assumes that whenever two hawks or two doves meet, both are equally likely to win. Therefore, their expected pay-offs are (*v* − *c*)/2 and *v*/2, respectively. If a hawk and a dove meet, the dove flees with zero gain while the hawk gets the whole resource *v*. Note that *c* > *v*, namely, the cost of conflict exceeds the value of the resource.

Now think in terms of the frequencies of strategies within a population. A homogeneous population of doves can be easily invaded by a few hawks, who win all contests without a fight. Conversely, a homogeneous population of hawks can also be invaded by a few doves. Although a dove has no gain from fleeing from a competitor, it is still better than the negative pay-off hawks get from escalated fights, which take place constantly in a nearly all-hawk population. Therefore, neither strategy alone is stable in the long run. Evolution will lead to a mixed population of both hawks and doves.

Maynard Smith & Price [4] laid out the logic of adaptation in such situations where the ‘environment’ consists of conspecifics and of the evolving population itself, and presented the definition of the evolutionarily stable strategy (ESS; box 1). However, on a much more general level (and largely powered by the influential and charismatic character of Maynard Smith: see [7]), the paper ignited the broad application of game-theoretical methodologies in evolutionary models. These applications have since diversified into a multitude of approaches. To a modern audience, the 1973 article of Maynard Smith and Price may not even be immediately recognizable as evolutionary game theory, with its computer simulations of games with a small set of discrete strategies. Leafing through the articles in this theme issue makes it apparent that the variety of methodological approaches in evolutionary game theory today is very broad, ranging from calculus-based study of continuous traits [11,19,20] to computer simulations [21,22]. Evolutionary game theory of continuous, quantitative traits also connects seamlessly with kin selection and modelling of evolution in class- and group-structured populations [11], and when we consider long-term evolution, evolutionary game theory is also relatively robust to complexities and constraints of the genetic system [8]. Furthermore, Lehtonen & Otsuka [19] argue that while the aim of evolutionary game theory models is typically to identify evolutionary endpoints, the mathematical components (partial derivatives and their combinations) arising in continuous, quantitative evolutionary games can themselves be useful in the causal interpretation of the evolutionary model and of fitness and selection, and that they can be interpreted in the ‘path coefficient’ formalism of Sewell Wright [23] and in the modern framework of causal modelling [24].

Box 1. **Stability criteria.**

Perhaps the single most influential concept introduced in Maynard Smith & Price [4] is that of the ESS. Explicit connections to game theory had been made in earlier evolutionary research [7–9], and the theoretical basis of sex ratio evolution was game-theoretical in nature before the concept of game theory even existed [5]. However, as a powerful theoretical idea, the ESS defined by Maynard Smith & Price [4] survives relatively unchanged to this day, at least on a conceptual level.

The ESS is analogous to (but slightly stricter than) a Nash equilibrium ([10] and box 1 in [6]). In the words of John Maynard Smith, an ESS (here denoted *I*) ‘must have the property that, if almost all members of the population adopt *I*, then the fitness of these typical members is greater than that of any possible mutant; otherwise, the mutant could invade the population, and *I* would not be stable’ ([2, p. 14]). Technical definitions differ depending on the application, and if one is unfamiliar with the field, it may not always be apparent that they refer to the same concept. In models of continuous, quantitative traits the ESS criterion is typically formulated in terms of a second derivative test which checks for local evolutionary stability (e.g. [11,12]). Phrased in terms of alleles, once an allele coding for an ESS value has reached fixation, no other mutant allele can increase in frequency (e.g. [13]). For technical definitions of ESS, we refer readers to [8], box 1 in [6], and [11].

In the case of a continuous strategy set, Maynard Smith ([2, p. 197]) noted that two conditions are needed (eqns 4 and 6 in [11], corresponding to the lack of directional selection, and to the ESS condition, respectively), but he missed a third one: in continuous games the ESS condition by itself is not quite enough. The ESS criterion tells us that once the population is at the ESS, other strategies cannot invade, but it says nothing about getting there in the first place, as noted by Eshel & Motro [14] (see also [15–17]). To ensure convergence towards the ESS, we also need the convergence stability criterion (eqn 5 in [11], box 1 in [6]). A less commonly used but related criterion is that of the neighbourhood invader strategy (NIS) [18]. Where convergence stability ensures that the strategy is attainable by successive, small mutations, NIS ensures that it is attainable by a single, fortuitous mutational jump.

Since the seminal work of Maynard Smith & Price [4], evolutionary game theory has become an indispensable tool for studying animal contests. Hardy & Mesterton-Gibbons [25] review the applications of evolutionary game theory as a testable theory framework and useful tool for studying contests, in particular, over host larvae by female parasitoid wasps. They present situations that closely match the dyadic owner-intruder contests and scenarios demonstrating winner–loser effects. The paper also discusses how game-theoretic modelling can be used to explore the application of the parasitoid wasps as biological control agents for crop pests, and the effect of synthetically produced agro-chemicals on their contest behaviours.

Evolutionary game theory also plays a key role in studying a variety of conflicting interests between the sexes. One example relates to the conflict between different optimal mating rates for females and males: Kovalov & Kokko [26] show that this conflict can be partly resolved if females develop fertility signals and males respond to them by targeting signalling females only. Furthermore, evolutionary game theory has been a successful modelling framework to elucidate the evolution of sex allocation [5] and the evolution of anisogamy [2,27]: while it is often presumed that the earliest anisogamous organisms had separate sexes, Henshaw *et al*. [21] present a model that examines the evolution of anisogamy in both monoecious (hermaphroditic) and dioecious (separate sexes) ancestral organisms.

Evolutionary game theory provides insight into the way humans and other animals make decisions in social interactions, and into the underlying mechanisms. Wang *et al*. [28] combine modelling and behavioural experiments with human participants to study public goods games with inequality in the participants' wealth or productivity. They find that a naïve application of the evolutionary models with random initial conditions often fails to produce the observed pattern in the experiments, while the accuracy of predictions remarkably improves once real-world initial conditions are implemented in the models. Also originating in the literature on human behaviour, Alger [29] reviews an emerging trend in economics research that extends the scope of game theory to evolutionarily stable preferences. In this growing literature, ‘strategies’ are treated as mere descriptions of behaviour, while ‘preferences’ incorporate mechanisms that underly different behaviours, such as reasoning, emotions, hormones and other neurobiological processes, which can respond to the (complete or incomplete) information that the individuals receive. Considering that in many cases nature selects the strategy choices of individuals not directly, but via their preferences, this emerging trend of research has the potential to strengthen the link between theory and reality.

In biological market interactions, individuals have multiple options to cooperate with a single or multiple partners, such as the interactions on the ‘mating market’ between conspecific males and females, between plants and pollinators, and between ants and lycaenid butterflies. Focusing on the biological market interactions in a marine cleaning mutualism between the marine cleaner wrasse *Laboroides dimidiatus* and its ‘client’ fishes, Bshary & Noë [30] review how the potential roles of cognition, the means of partner coercion, and the interactions between partner quality and the supply-to-demand ratio affect service quality. While biological market models are generally designed to focus on interactions within a constant environment, evolutionary game theory has been applied to study animals' decision-making across different environments. Shaw *et al*. [31] review the application of evolutionary game theory in studies on seasonal migration and present a new model to study whether infection by a novel parasite in a different environment may lead to migration loss. A promising way to connect studies in humans and other animals is to use the comparative economics approach (reviewed in [32]) by which humans and other animals (usually primates) are put into identical contexts for making decisions. This approach has revealed great variation in the ability to make ‘optimal’ decisions across species and different underlying proximate mechanisms that lead to the same decision-making outcome.

While evolutionary game theory models are generally constructed to focus on the fitness consequences of social interactions, results from studying social interactions in humans and other animals as well as those revealed by comparative economics studies show that there is much potential in integrating the proximate and ultimate aspects of social behaviours in future evolutionary game theory models. Kuhn *et al*. [22] combine simulations and microbiology experiments to incorporate ecological factors such as the flows of nutrients and toxin in the environment into the ‘rock-paper-scissors’ evolutionary game models. They use three-dimensional-printed bioreactors to keep the competition between three *Escherichia coli* strains in continuous culture while preserving their spatial distribution. By tracking the frequency changes of the three strains over two weeks, they found that the evolutionary dynamics differ greatly from the cyclic dominance pattern predicted by classical evolutionary game theory models, but can be explained by simulations that consider the relevant ecological factors.

Evolutionary game theory is a versatile modelling framework that can be naturally connected to other major modelling approaches, such as the Lotka–Volterra equations commonly used in ecology, and the Wright-Fisher or Moran process models frequently used in population genetics. Traulsen & Glynatsi [33] present a critical review of the role of evolutionary game theory in connecting evolutionary biology and other disciplines including physics, mathematics, economics and computer sciences, as well as the cross-fertilization and sometimes parallel development of similar ideas in different subfields or research topics of evolutionary game theory. The authors also point out several problems that have been limiting the development of the field, such as an over-emphasis on a small number of popular research questions and the lack of mutual understanding and crosstalk between theoreticians and empiricists. Because of the interdisciplinary nature of evolutionary game theory, it has the potential to be applied widely to conceptualize and help solve real-world problems. Stein *et al*. [20] review the many applications of Stackelberg game theory in steering evolving systems, such as fisheries management, cancer treatment and pest control. In Stackelberg games, there are ‘leaders’ and ‘followers'. Traditionally in economics, these were companies where the leader firm moves first, and then the follower firms move sequentially, but in evolutionary game theory, both leader and follower can take diverse forms. In the examples reviewed in [20], humans play the role of a rational leader and the systems to be managed respond as evolving followers. The authors show that the leader can often obtain the most profitable result by anticipating and steering the eco-evolutionary dynamics accordingly.

Evolutionary game theory has come a long way since it was first hinted at in models of sex allocation [5] and since it was elevated to its status as a central part of evolutionary theory by Maynard Smith & Price [4] (see [6,7]). It has become an essential tool in the evolutionary modeller's toolkit [34] and has been tightly linked with a range of theoretical concepts and methods [11]. We hope this special issue sheds light on the range of applications that evolutionary game theory has today, and on the range of research that it has inspired—and that this collection of articles will perhaps inspire the next generation of evolutionary game theorists.

## Data Availability

This article has no additional data.
